# Postoperative Pain Relief With Ultrasound-Guided Dorsal Sacral Foramen Block for Foot and Ankle Surgeries

**DOI:** 10.7759/cureus.22701

**Published:** 2022-02-28

**Authors:** Sandeep Diwan, Madhuri Dadke, Avinash Gaikwad, Himaunshu Dongre, Ganesh P Bhong, Parag K Sancheti, Abhijit Nair

**Affiliations:** 1 Anaesthesiology, Sancheti Institute for Orthopaedics and Rehabilitation, Pune, IND; 2 Orthopaedics and Trauma, Sancheti Institute for Orthopaedics and Rehabilitation, Pune, IND; 3 Anaesthesiology, Ibra Hospital, Ibra, OMN

**Keywords:** sacral epidural space, popliteal nerve block, postoperative pain, ultrasound guided regional anesthesia, ultrasonography, sacral foramen, elective foot and ankle surgeries

## Abstract

This case series describes the use of ultrasound (US)-guided dorsal sacral foraminal block (DSFB) for providing postoperative analgesia in six patients who underwent foot and ankle surgeries under spinal anesthesia. Postoperatively, all of them received a US-guided DSFB at the level of the brim of the second sacral foramina (SF2). Needle placements were confirmed with fluoroscopic (FL) images and injected radiocontrast defined the diffusion with a postoperative CT scan. The images obtained depicted ipsilateral spread in the sacral epidural space, sacral nerve roots, and plexus. The US-guided DSFB could be effectively used as an alternative method for postoperative pain relief after foot and ankle surgery.

## Introduction

The popliteal sciatic nerve block (PSNB), combined with the adductor canal block or the femoral nerve block (FNB), is recommended for postoperative analgesia in foot and ankle surgery [1,2]. Apart from multiple puncture points, no permanent neuropathic effects have been reported with PSNB. The recent trend is to avoid the injection in the vicinity of the nerve or the neuraxial spaces, and access the nerves traversing the inter-muscular, interfascial, or the bone muscle-ligament interfaces, which innervate similar dermatomes as the former (e.g., the quadratus lumborum or the transversus abdominis planes instead of neuraxial space). With a drift from the neuraxial space to interfascial planes, it is expected that the analgesic effect might not be equivalent but should not be inferior either [3]. As in the thoracic erector spinae plane block, indirect access to the paravertebral and the neuraxial space through the existing opening would be a viable alternative. Similar to thoracic erector spinae injections where the costotransverse foramina offer a conduit to ventral translocation of local anesthetic (LA), we hypothesized that injection at the brim of the sacral foramina offers a potential passage between the dorsal and ventral aspects of the sacrum. Several anatomical landmark-based and fluoroscopic (FL) guided approaches of injections into the second dorsal sacral foramen (DSF2) for sacral spinal and epidural injection have been described. We present a series of six patients undergoing foot and ankle surgery who received ultrasound (US)-guided dorsal sacral foramen block (DSFB) at the level of the brim of the second sacral foramen (SF2). The technique was coupled with FL confirmation of the needle position in anteroposterior (AP) and lateral views and post-procedural CT scan to map the spread of the injected radiocontrast.

## Materials and methods

Ethical approval was obtained from the Institutional Ethical Committee (dated March 11, 2021) to study six patients with the American Society of Anesthesiologists’ physical status (ASA-PS) I and II. There were four males (aged 24, 36, 54, and 49 years), two females (aged 22 and 63 years) who were scheduled to undergo foot and ankle surgeries (n=2: Achilles tendon rupture; n=2: fractures of calcaneum; n=2: subtalar arthrodesis). All patients received spinal anesthesia with 3 ml of 0.5% heavy bupivacaine as the primary anesthetic. Immediately after the surgical procedure in the corresponding position [prone position in two patients (Achilles tendon repair) and lateral position in four patients (fractures of the calcaneum and subtalar arthrodesis)], DSFB was administered. The surgery for Achilles tendon repair was performed in the prone position, and hence the block was executed in a similar position. However, the surgeries for calcaneum and talus were in lateral position with operated side non-dominant and the dorsal foraminal block was implemented in a similar position. Written informed consent was obtained for postoperative CT contrast studies for all patients.

US-guided DSFB technique

**Figure 1:**
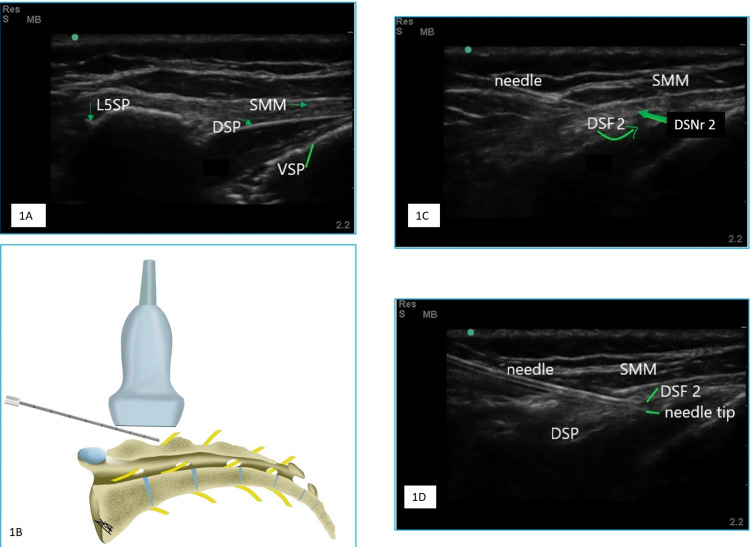
Images showing relevant sonoanatomy and probe placement 1A: Ultrasound long-axis image at the fifth lumbar spinous process (L5 SP), depicting the dorsal sacral plate (DSP) beneath the sacral multifidus muscle (SMM), the ventral sacral plate (VSP), and the plausible sacral epidural space (SES) 1B: Linear probe at the lateral to intermediate crest depicting the emergence of the dorsal sacral nerves (DSNr) and needle-tip placement at the brim of dorsal sacral foramina (DSF) 1C: Needle tip positioned at the brim of the second dorsal sacral foramina (DSF2), proximal to the emergence of DSNr2 1D: The needle tip at the brim (outer border of the DSF) of the DSF2, slow injection of local anesthetic

After antiseptic preparation, a 5-13 MHz linear array US probe (M-turbo, Fuji Sonosite, Bothell, WA) was deployed sagittally over the L5-S1 spinous process (Figure 1A). With a lateral shift of the probe on the dorsal aspect of the sacrum, the intermediate crest was identified. A slight caudal and lateral shift to the intermediate crest revealed the break in the continuity of the dorsal sacral plate (Figures 1B, 1C). The first aperture was considered the DSF2. Caudal to the DSF2, the DSF3 was noted. Hypoechoic structures (sacral nerve roots) were seen emerging from both the apertures and coursing caudally (Figure 1C). The Color mode did not detect vascular structures in the vicinity of the dorsal sacral nerve roots. A 21 G 100 mm short bevel (B-Braun, Melsungen, Germany) insulated needle was inserted in-plane from the cephalad to the caudal direction (Figures 1B, 1D). A loss of resistance could be appreciated as the tip penetrated the fascial layer covering DSF2 and the tip was positioned at the brim of DSF2 (Figure 1D).

Fluoroscopy technique

**Figure 2:**
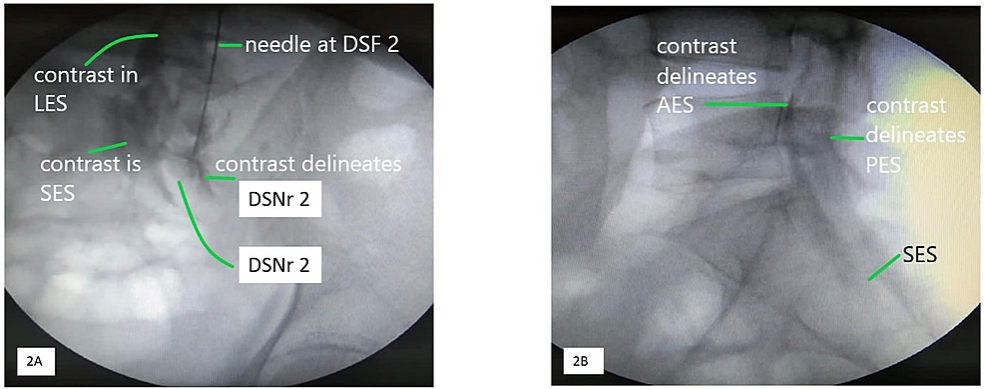
Structures seen on fluoroscopy 2A: Fluoroscopic anteroposterior image depicting the needle tip at the second dorsal sacral foramina (DSF2) and contrast spread in the lower lumbar epidural space (LES) and upper sacral epidural space (SES). Contrast delineates the second dorsal sacral nerve root (DSNr2) 2B: Lateral view depicting the contrast delineating the anterior and posterior epidural space (AES and PES) respectively. The contrast extends into the SES

The AP and lateral fluoroscopic images were obtained to confirm the needle-tip position and exclude the needle tip in the sacral epidural space (Figure 2A). Following negative aspiration for blood and CSF, 5 ml of 2% lidocaine with 1:200,000 adrenaline was injected as a test dose. With no change in vital signs, 5 ml of Omnipaque (Iohexol 300 mg iodine/ml) and 15 ml of 0.2% ropivacaine were injected slowly in small 5 ml incremental boluses. FL AP and lateral views were repeated after the injection to assess the extent of the spread of the solution. Contrast delineated the ipsilateral DSF2 with a unilateral spread in the epidural space in the AP view. The lateral view illustrated contrast in anterior and posterior epidural spaces (Figure 2B). Patients were turned supine and monitored for 15 minutes before they were shifted to the recovery room. CT scan was performed at the first hour after the block.

## Results

**Figure 3:**
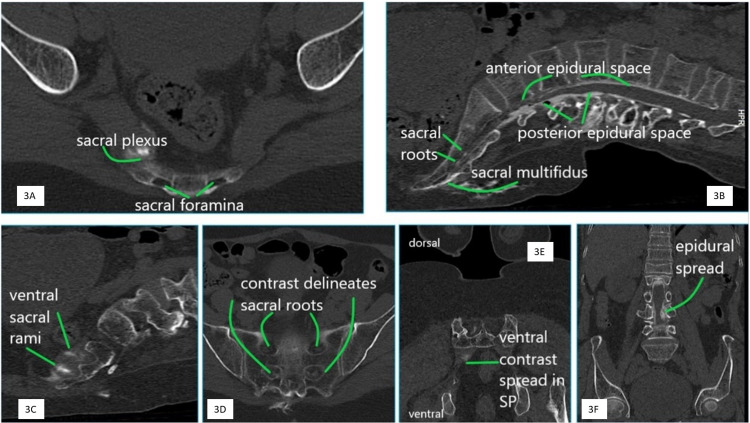
CT contrast images 3A: Axial section at the SF3 level demonstrating the foramina and spread of the contrast in the sacral plexus 3B: Sagittal view depicting the contrast-stained sacral roots and delineation of the anterior and posterior epidural space until the upper portion of L1. Contrast delineates the sacral multifidus muscle and spills into the dorsal fat tissue 3C: Sagittal section with contrast delineating lower ventral sacral rami 3D: Coronal view illustrates contrast-delineated bilateral sacral roots in the sacral foramina at the levels S1 and S2 CT: computed tomography; SF: sacral foramen

In the postoperative period, all patients were monitored for vital parameters like heart rate, blood pressure, oxygen saturation, and pain scores at zero, six, 12, 18, 24, 36, and 48 hours. CT scan was performed at the first hour after the block. CT contrast studies (Figures 3A-3F) revealed a spread in the epidural space (6/6) from sacral to the lumbar in all planes (6/6), staining the sacral nerve roots (6/6) and contrast emerging in the pre-sacral area (2/6). The extent of the epidural spread in axial, sagittal, and coronal planes is briefed in Table 1. A unilateral contrast diffusion occurred in the sacral multifidus plane in all patients.

**Table 1:** Extent of epidural spread SP: sacral plexus; Y: yes; N: no

	SP	S4	S3	S2	S1	L5	L4	L3	L2	L1	T12	Extent
Axial												
Case 1	Left	Y	Y	Y	Y	Y	Y	Y	Y	Y	Y	S4-T12
Case 2	N	N	Y	Y	Y	Y	Y	Y	N	N	N	S3-L3
Case 3	Right	Y	Y	Y	Y	Y	Y	N	N	N	N	S4-L3
Case 4	N	N	Y	Y	Y	Y	Y	Y	Y	Y	Y	S3-T12
Case 5	N	N	Y	Y	Y	Y	Y	Y	Y	N	N	S3-L2
Case 6	N	N	Y	Y	Y	Y	Y	Y	Y	N	N	S3-L2
Sagittal												
Case 1	Left	Y	Y	Y	Y	Y	Y	Y	Y	Y	Y	S4-T12
Case 2	N	N	Y	Y	Y	Y	N	N	N	N	N	S3-L5
Case 3	Right	N	Y	Y	Y	Y	Y	Y	Y	N	N	S3-L2
Case 4	N	N	N	Y	Y	Y	Y	Y	Y	Y	Y	S2-T12
Case 5	N	N	N	Y	Y	Y	Y	Y	Y	Y	N	S2-L1
Case 6	N	N	Y	Y	Y	Y	Y	Y	Y	N	N	S2-L2
Coronal												
Case 1	Left	Y	Y	Y	Y	Y	Y	Y	Y	Y	Y	S4-T12
Case 2	N	N	Y	Y	Y	Y	Y	Y	N	N	N	S3-L3
Case 3	Right	Y	Y	Y	Y	Y	Y	N	N	N	N	S4-L4
Case 4	N	N	Y	Y	Y	Y	Y	Y	Y	N	N	S4-L2
Case 5	N	N	N	Y	Y	Y	Y	Y	Y	N	N	S2-L2
Case 6	N	N	N	Y	Y	Y	Y	Y	Y	N	N	S2-L2

The pain scores were monitored using a numerical rating scale (NRS) at zero, six, 12, 18, 24, 36, and 48 hours from the block (Table 2). All patients received intravenous (IV) paracetamol 1 gm eight hourly and continued 12 hourly. When the NRS score was reported to be 4 or more, 50 mg IV infusion of tramadol was administered as rescue analgesia. At the sixth hour of post-regression of spinal anesthesia, the sensory evaluation revealed unilateral complete blunting of sensations to light pinprick in the region innervated from L1-S3 and patchy numbness on the contralateral side in the area from S1-3. However, motor functions (quadriceps and ankle movement) were intact.

**Table 2:** Pain scores and time to the first analgesic ORIF: open reduction, internal fixation

Patient number	Surgery	Pain scores								
		0 hours	6 hours	12 hours	18 hours	24 hours	36 hours	48 hours	Rescue analgesia time	Number of doses
1	Left Achilles tendon repair	0	1	3	2	2	1	1	--	0
2	Left calcaneum fracture ORIF	0	2	1	1	2	1	2	14	1
3	Right subtalar arthrodesis	0	3	2	2	1	2	1	08	2
4	Left Achilles tendon repair	0	2	1	2	2	1	2	--	0
5	Left subtalar arthrodesis	0	2	2	4	4	3	1	08	1
6	Right calcaneum fracture ORIF	0	2	2	4	4	4	1	06	2

## Discussion

US identification of DSF2 and needle-tip placement at the brim of the sacral dorsal foramina was possible in all patients and was confirmed with fluoroscopy. Diffusion of LA injections could be visualized in real-time through the acoustic window of DSF2 and DSF3 into the sacral epidural space. CT radiocontrast images demonstrated a diffusion from sacral to lumbar epidural space and a spread in the ipsilateral sacral plexus. No significant spread was identified in the sacral multifidus plane.

The initial epidural cephalad spread was possible because of (1) the larger internal width of upper sacral epidural space accommodating more LA than the narrower lower sacral epidural space and (2) the topographical arrangement (curvature from point of injection at DSF2) of the sacral to lumbar epidural space. Two patients with medial incision (dermatome L2-4) for subtalar arthrodesis had effective postoperative analgesia signifying adequate blockade in the saphenous nerve distribution (L3, L4). Foot and ankle innervated by neural elements of lumbar (anterior primary rami of spinal nerves T12 to L5) and sacral plexus (anterior primary rami of spinal nerves L4, 5, S1-4). The saphenous nerve, a continuation of the femoral nerve in the proximal thigh, innervates the periosteum of the tibia and the medial malleolus and articular branch terminate into the capsule of the calcaneonavicular joint. The tibial nerve, through its medial calcaneal branches, merges into the dorsal aspect of the ankle joint and extends up to the talocalcaneonavicular joint. The superficial peroneal and deep peroneal nerves innervate the ventral tibiofibular joint and lateral and ventral aspects of the capsule of the ankle joint and the talocalcaneonavicular joint respectively. The sural nerve merges within the periosteum of the fibula and innervates the capsule of the talofibular and the talocalcaneal joints and, along the lateral edge of the foot, innervates the subtalar and the talonavicular joints. The cutaneous branch of the saphenous nerve terminates on its medial aspect at the medial malleolus; however, it merges with the capsule of the first metatarsal [4]. The dorsum of the foot is chiefly innervated by the superficial peroneal nerve, the lateral and posterior by the sural, and the plantar surface by the medial and lateral plantar nerves from the tibial nerves [5].

Based on anatomical landmarks, FL guidance, and cadaveric anatomic study, a presacral nerve block [6], trans-sacral epidural anesthesia [7], sacral spinal anesthesia [8], and sacral nerve stimulation [9] have been described. Anatomical variations [10,11,12] and FL-related harmful radiation could probably hinder the future implementation of these modalities.

The US is a non-radiation imaging modality, which provides a safe and reliable method to identify the anatomical landmarks, the sacral foramina, and the dorsal sacral roots with their accompanying vessels [13]. The width and the inter-foraminal distances of DSF are 0.76 cm and 1.3 cm respectively [14]. Thus, it is plausible for LA to diffuse through multiple DSF. Therefore, we recommend a slow rate of injections at 5 ml every 15 seconds with a waiting period of five seconds. We used fluoroscopy in our series to register the accuracy of the US technique and obtained CT scan images to document the spread of the injectate. Both of these imaging modalities will not be necessary in the future to confirm US-guided needle placement and spread of LA. 

Patients were exposed to fluoroscopy and CT scan, a radiation hazard, but were appropriately counseled for the same. Paraesthesia suggesting a needle-to-nerve contact could not be elicited, since the DSFB was performed under the effect of spinal anesthesia. Additionally, we did not possess the equipment to monitor the intraneural pressures.

## Conclusions

DSFB is an alternative technique and it provided effective postoperative analgesia after foot and ankle surgeries in all of our patients. Based on FL and CT contrast images, we presume that the DSFB is an indirect approach to neuraxial space in the sacral area. However, it would be technically challenging for a novice to execute. Hence, we described a potential non-radiation, non-neurostimulation US-guided DSFB at the SF2 level. However, further studies are needed to explore its role in lower extremity surgeries innervated by the lumbosacral plexus.
